# Mining Unique-*m* Substrings from Genomes

**DOI:** 10.4172/jpb.1000127

**Published:** 2010-03-16

**Authors:** Kai Ye, Zhenyu Jia, Yipeng Wang, Paul Flicek, Rolf Apweiler

**Affiliations:** 1 Molecular Epidemiology section, Medical Statistics and Bioinformatics, Leiden University Medical Center, The Netherlands; 2 Department of Pathology & Laboratory Medicine, University of California, Irvine, CA 92697, USA; 3 Vaccine Research Institute of San Diego, San Diego, CA 92121, USA; 4 EMBL Outstation, European Bioinformatics Institute (EBI), Wellcome Trust Genome Campus, Hinxton, Cambridge, UK

**Keywords:** Data mining, Genomes, Mismatch, Sequence

## Abstract

Unique substrings in genomes may indicate high level of specificity which is crucial and fundamental to many genetics studies, such as PCR, microarray hybridization, Southern and Northern blotting, RNA interference (RNAi), and genome (re)sequencing. However, being unique sequence in the genome alone is not adequate to guaranty high specificity. For example, nucleotides mismatches within a certain tolerance may impair specificity even if an interested substring occur only once in the genome. In this study we propose the concept of unique-*m* substrings of genomes for controlling specificity in genome-wide assays. A unique-*m* substring is defined if it only has a single perfect match on one strand of the entire genome while all other approximate matches must have more than *m* mismatches. We developed a pattern growth approach to systematically mine such unique-*m* substrings from a given genome. Our algorithm does not need a pre-processing step to extract sequential information which is required by most of other rival methods. The search for unique-*m* substrings from genomes is performed as a single task of regular data mining so that the similarities among queries are utilized to achieve tremendous speedup. The runtime of our algorithm is linear to the sizes of input genomes and the length of unique-*m* substrings. In addition, the unique-*m* mining algorithm has been parallelized to facilitate genome-wide computation on a cluster or a single machine of multiple CPUs with shared memory.

## Introduction

Unique substrings in genomes may indicate high level of specificity which is crucial to many genetics studies, such as PCR, microarray hybridization, Southern and Northern blotting, RNA interference (RNAi), and genome (re)sequencing. However, being unique sequence in the genome alone is not adequate to guaranty high specificity. For example, in microarray studies, a small fragment of DNA probe is used to fish for particular RNA/DNA molecules in the test sample. This probe will capture those RNA/DNA molecules that contain a perfect reverse-complement counterpart. Albeit with relatively lower hybridization intensity, molecules with a couple of mismatches can also be caught by the probe, which is called cross-hybridization and therefore reduces the probe specificity. We consider a DNA fragment as a poor probe if it has quite a few approximately matched sequences in the genome. Those approximate matches can be very confounding since we are uncertain which RNA/DNA molecules in the sample does the probe represent. The chance of RNA/DNA being fished by the probe drops dramatically as the number of mismatches increases. Thus we may use the number of mismatches as an indicator for the probability of cross-hybridization for a probe if we ignore the impact of the distribution of the mismatches along the probe sequence.

In this study we define a DNA fragment as a unique-*m* probe if it perfectly matches exactly one spot on one of the two strands of the genome while any of its approximate matches along the genome have more than *m* mismatches. In this way we have a solid criterion to design appropriate DNA probes for Microarrays. Similarly, we might also use these unique-*m* substrings to design preferable siRNA to reduce off-target effects. As for genome re-sequencing efforts, we may use the information about unique-*m* substrings to interpret and evaluate the mapping results of short reads. Those reads that meet with unique-*m* properties (*m* > 2) are trusted with higher confidence than others which match other locations of the genome with one or two mismatches. Last but not least, with the exponential growth of sequence databases, there is tremendous need to develop techniques to mine and output unique-*m* substrings and their locations in genomes in an effective way, e.g., linear in time and space.

The data structures of hash tables and suffix arrays were previously used to find (minimum) unique substrings of the human genome for the whole genome tiling arrays (Bertone et al., 2006; Graf et al., 2007). Bertone et al applied BLAST-like scheme: sequences in the database are preprocessed into a hash table of *k*-tuples so that the potential locations of a query can be efficiently narrowed down by looking for the *k*-tuples in the query sequence from the hash table (Altschul et al., 1990; Ning et al., 2001). As for suffix arrays, they can be built linearly in time and space, given sufficient memory. Several algorithms have been proposed to build suffix trees in memory efficiently (Edward 1976; Giegerich and Kurtz, 1997; Ukkonen, 1995; Weiner, 1973), but they do not scale up well due to huge space consumption (about 5-16 times of the input database) when the input databases approach the size of the human genome (Publisi et al., 2007).

To overcome aforementioned obstacles, we provide a pattern growth scheme, a data mining oriented algorithm, which guarantees to find all unique-*m* substrings of genomes linearly in time and space. Our algorithm traces both perfect and approximate matches with no more than *m* mismatches on both strands of the genome sequences where the value of *m* is provided by the user. The divide-and-conquer strategy is readily incorporated into the mining process so that the eventual calculationcan be allocated either over multiple CPUs with shared memory or on a cluster.

## Methods

### Algorithms

The inputs of the algorithm are a dataset *S* which consists of a series of non-empty sequences of the alphebet {A, C, G, T}, a minimum (*min_l*) as well as a maximum (*max_l*) length of substrings to search for and the number of mismatches *m*.

The output of the algorithm consists of all substrings that appear exactly once in one of the two strands of the input sequences and all the other approximate matches must have more than *m* mismatches. The locations of those unique-*m* substrings will be given from implementing the algorithm.

The algorithm is as follows. Let *a* be a substring and *S_a_* be the so-called projected database that contains all sequences that contain the substring *a*, where the last element of each occurrence of *a* is marked (the so-called *a*-locations) in both strands of the sequences and the number of mismatches for each occurrence is also recorded. The computation of *S_a’_* from *S_a_* requires, for each *a*-location, the check of whether or not the base on its right side (if any) equals the newly appended item b. In case of equality this gives an *a’*-location in *S_a’_* with the same number of mismatches; otherwise one more mismatch will be logged. If the number of mismatches exceeds *m*, this *a’*-location is not stored.

   *unique-m* (a, S_a_)

      if |*S_a_* | ≥ 1 then

         if *min_l* ≤ *length* (*a*) ≤ *max_l* and |*S_a_* | = 1 then

               report *a* and *S_a_*

      if *length* (*a*) < *max_l*

         for each base b do

                       *a’* ≔ *a* with b appended to it

                       *unique-m* (a’, S_a’_)

The main call is *unique-m* (Λ, *S*Λ), where Λ is the empty substring. Note that each base in database *S* Λ is a Λ–location, including the position *before* each sequence (with *ϕ* mismatch). This call creates a projected database that marks all occurrences of the first base b, reports all unique*-m* substrings that begin with this b, and then proceeds to the next base.

### Datasets

To evaluate our unique*-m* substring mining program, we collected the whole “genome” sequences of various “species” with different sizes. The smallest genome in this study is the genome of the Phi-X 174 bacteriophage while the human genome is the largest, as listed in Table 1.

### Computer

Both single and multi-threaded tests were performed on the same PC configured with 8 processors of Intel Xeon E5420, 8GB of RAM and 250GB hard drive. In benchmarking studies for single thread, we implemented the program to run in serial mode so that only one core was used.

## Results

### Implementations of the unique-*m* substring mining algorithm

The unique-*m* substring mining algorithm was implemented in C++ and it has been multithreaded with openMP (www.openmp.org, a shared memory parallelism). User needs to input genome sequence, the length of unique-*m* substrings, the maximum number of mismatches (*m*), and may also specify the number of threads to operate when executing the program.

### Runtime of mining unique substrings is linear to the input size

**Figure 1:**
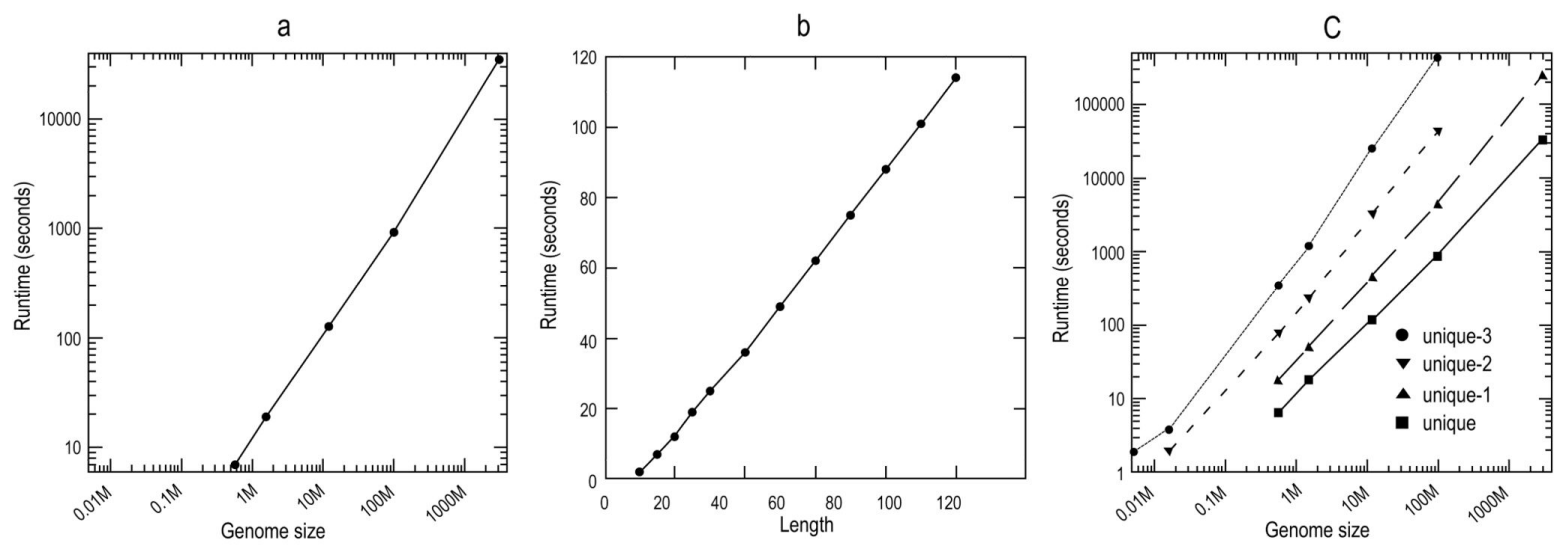
Linear correlation between the runtime and the size of the input genome. **a:** The runtimes in mining unique 25-mers in various “genomes”. **b:** The runtimes in search for unique n-mers (10 = n = 100) in the genome of Thermoplasma acidophilum (about 1.5 million bases). **c:** The runtimes of mining unique*-m* substrings in various “genomes”. Note that only unique and unique-1 25-mers were computed for human genome. The *x* and *y* axes in **a** and **c** are logarithmically scaled.

As mentioned in the Method section, our program first initializes a projected database for a given nucleotide or substring and then recursively extends the substring and mines the associated projected databases. Theoretically, the size of the projected database and the number of recursive mining operations are proportional to the size of the input sequences, which has been versified in our first experiment. We ran our unique*-m* program to mine unique 25-mers using various “genomes” as test sets. As shown in Figure 1a, if the genome size was smaller than 1 million bases, the runtime was a few seconds only. When the genome size reached 100 million bases (C.elegans), it took about 15 minutes to find all unique 25-mers. As for the human genome which is more than 30 times that of C.elegans’s, the entire computation was completed within 10 hours.

### Runtime for mining longer unique substrings

It is apparent that when we look for longer substrings, the number of potential substrings (4^n^) to examine will increase exponentially. However, as shown in Figure 1b, for our algorithm the runtime is still linear to the length of substrings (from 10 to 100) because the number of survived locations in the genome decreases exponentially in each extension step. The genome of Thermoplasma acidophilum (about 1.5 million bases) was used to search for all unique *n*-mers for demonstration.

### Search for unique-*m* substrings in genomes

As for the unique-*m* substrings, they will only match one position of the genome and all the other approximate matches will have more than *m* mismatches. Apparently, by increasing the *m* values, we will be able to identify probes that have improved uniqueness in genome-wide assays. However, the increase in *m* compensates for computational expenses since more variants of a substring and more locations in the genome need to be considered with extended substrings.

**Figure 2:**
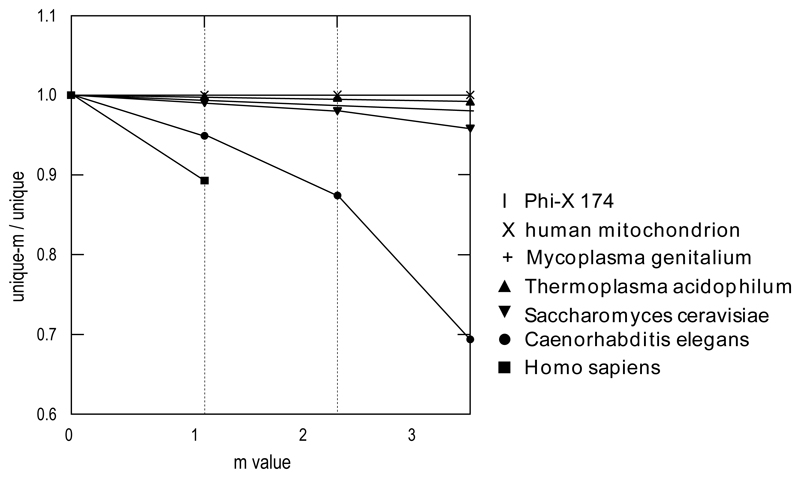
The ratio of unique*-m versus* unique 25-mers in various genomes. The numbers of unique 25-mers were computed first. Then unique*-m* 25-mers were identified for the seven species. We increased the *m* value up to 3 while we only computed the *unique-*1 25-mers for the human genome. Finally the ratio of unique*-m versus* unique in each genome was plotted against the *m* value.

As shown in Figure 1c, for any given genome, the runtime of mining unique*-m* substrings indeed increases exponentially as we increase the value of *m*. However for any particular *m* value, the runtime is still linear to the size of input genome. The number of unique*-m* substrings in a particular genome shrinks with the increase of the *m* value (Figure 2). Such phenomenon tends to be more profound in larger genomes. For C.elegans, about 5% of the unique 25-mers match another location on the genome with a single mismatch, and more than 30% with no more than 3 mismatches. As for the human genome, 10% of the unique 25-mers will match another location of the genome.

### Parallelization

As major CPU vendors are encountering many technological problems in boosting speeds on single CPU, they have shifted gears away from ramping up clock speeds to adding parallelism with multicore processors to offer a better-performing processor. In this study, the unique-*m* algorithm is parallelized with openMP (http://www.openmp.org/, a shared memory parallelism) and runs on machines configured with multi-CPUs. As explained in the Method section, we first initialized a projected database to store the locations of a particular substring in a genome and then recursively extended this substring while updating the projected database. When we initialize the projected database with the 1-mers, the computation is then readily divided into four subroutines for the four nucleobases. We could split the computation further by initializing the projected databases with longer substrings. In this case, the number of subroutines is 4^n^ where *n* is the length of the substrings.

**Figure 3:**
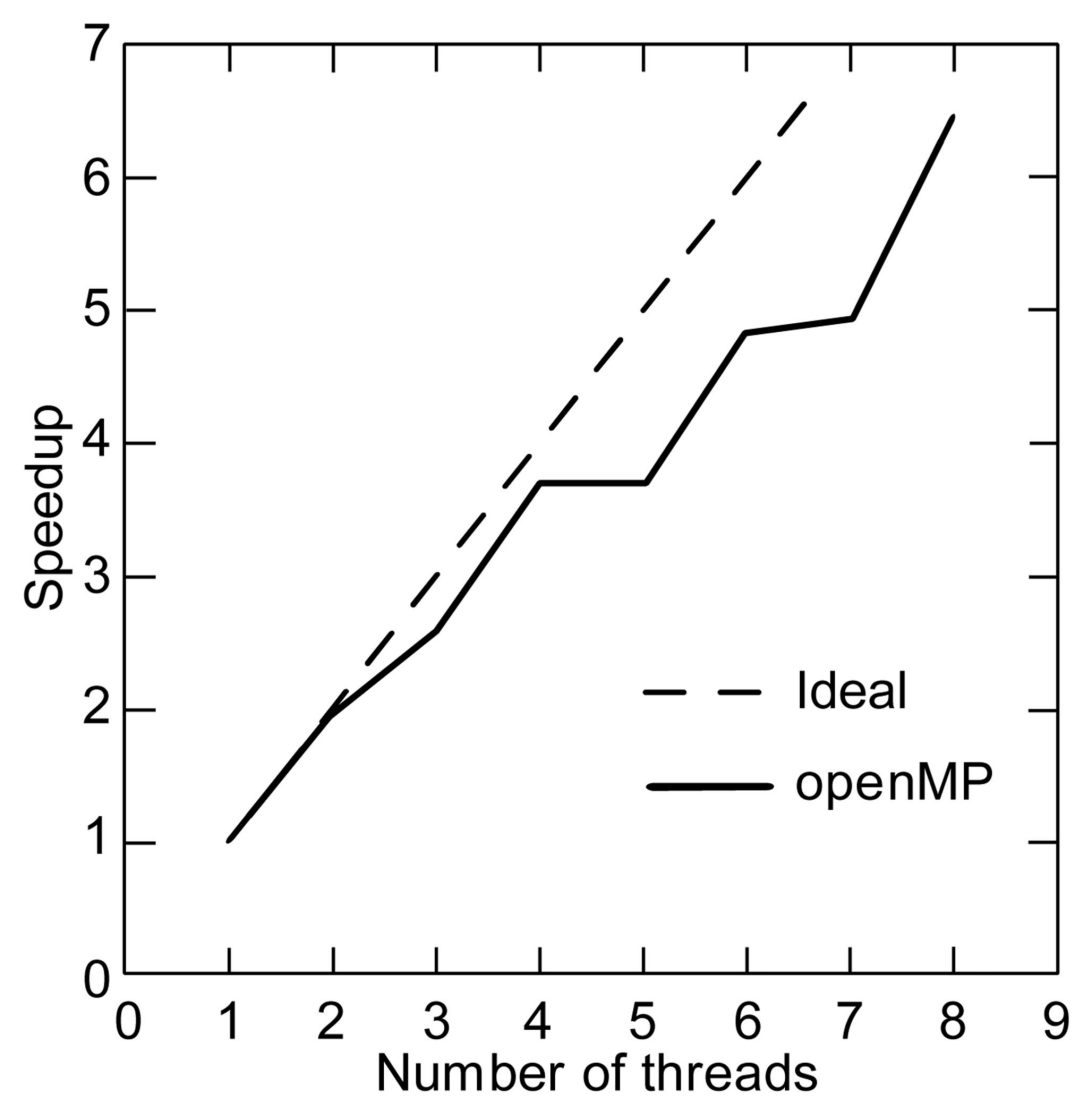
Parallel speedup using openMP. The genome of Thermoplasma acidophilum (about 1.5 million bases) was used compute unique-2 12-mers. The projected databases are initialized with 3-mers so that a pool of 64 processes was available for parallelization.

As shown in Figure 3, the program shows excellent speed up with openMP because each subroutine is completely independent so that there is little synchronization among different threads.

The source code of our unique*-m* mining can be compiled using Intel C++ compiler to run on a cluster. Alternatively, the calculation can be divided into multiple jobs according to prefixes and then run all of them on a cluster. For example, if the user specifies a prefix “ACG”, our program will only search for the unique*-m* substrings with this particular prefix.

## Discussion

### Pattern growth reduces search space

A straightforward algorithm to find all unique*-m k*-mers from genomes is to generate and test all possible *k*-mers. It is not difficult to estimate the number of 25-mers to be examined in such an approach. Since at each position all four nucleotides may occur, the total number of 25-mer candidates would be 4^25^ ˜ 10^15^. Moreover, for each 25-mer candidate, the entire genome sequences would have to be scanned to record the locations of matches with up to *m* mismatches. Thus, a sophisticated method is demanded.

In this study, we present a pattern growth solution to remarkably reduce the number of *k*-mers and the cost of multiple scanning of the dataset. We grow a given substring by adding elements to its right-hand side. Once the new substring does not appear in both strands of the genome, we stop growing it and its extension will not be tested. For example, suppose a string AGCCGAT does not have any matches in both strands, four possible extensions, AGCCGATA, AGCCGATC, AGCCGATG and AGCCGATT won’t be subjected to any further test. In this way, the number of *k*-mer candidates examined is significantly reduced while all *k*-mers that satisfy the input requirement will be reported.

In addition, this pattern growth method significantly reduces the efforts in scanning the entire dataset. If we want to examine the locations of a particular string in the genome, the algorithm does not scan the whole genome but only checks the locations where its prefix is, recursively.

### Memory requirement

In the present prototype of unique*-m* substring mining algorithm, we have not fully optimized its speed and memory usage. For example, the genome is loaded into the memory without compressing the bases into bits. We used unsigned integers in C++ to record the locations of a given substring in the projected databases. We may consider using relative distances to anchor points instead of absolute locations to save space in the projected databases.

In the current setting, our program still utilizes much less space than suffix trees or suffix arrays algorithms in finding unique substrings in genomes. Let us consider the average memory usage if we assume that the numbers of occurrences for four bases are comparable in the genome. We take the size of the input genome as the unit. In this case, we first spent one unit of memory to store the input genome. Then for each of the four bases, we created a projected database to record the locations of this base on both strands. We used 32 bits integers in C++ to index the locations. Thus we need two units of memory to store such projected database. After that, for each extension of the substring, the projected database is about 25% of the previous one. Thus the total memory consumption of mining all unique substrings with any given length *n* is

$$1+2+2*{\left(\frac{1}{4}\right)}^{1}+2*{\left(\frac{1}{4}\right)}^{2}+\ldots +2*{\left(\frac{1}{4}\right)}^{n}$$

which converges to if *n* approaches infinity.

We may also initialize the projected database with a longer prefix to save memory consumption. For example, if we had used *k*-mers instead of the 1-mers to initialize the projected database, the memory consumption is

$$1+8*[{\left(\frac{1}{4}\right)}^{k}+{\left(\frac{1}{4}\right)}^{k+1}+\ldots +{\left(\frac{1}{4}\right)}^{n}]$$

which converges to

$$1+\frac{2}{3}*4^{2-k}.$$

In this case, if we use 2-mers to initialize the projected database, we only need 5/3* 3GB = 5GB of memory to find unique substrings of any length from the human genome. Note that 3 GB out of 5 GB is used to store the sequences of the human genome.

On the other hand, if we want to mine unique*-m* substrings instead of unique ones, the memory consumption is

$$1+\frac{2}{3}*4^{m+2-k}.$$

### Approaches to improving oligonucleotide probe specificity

A variety of approaches have been routinely implemented for estimating the specificity of oligonucleotide probes in microarray platform design (Nordberg, 2005; Chen, 2002; Reymond, 2004), including (i) removing probes with more than 75% identity with a non-target sequence (Hughes et al., 2001; Kane et al., 2000), (ii) removing probes that include contiguous stretches (more than 15nt) of identical sequences with a non-target sequence (Kane et al., 2000), (iii) removing probes with low complexity regions, such as long stretches of the same base (i.e. “AAAAAA” or “TTTTTT”). We recently found that there is still a lot of room to improve the probe specificity although great endeavor had been made to do so (Xia, in press). Unique-*m* substring approach provides a unique way to control for the probe specificity. The number of mismatches (*m*) should be customized for different microarray studies which vary in probe length, sample species and microarray platform etc.

## Conclusions

In this study we propose the concept of unique-m substrings of genomes for controlling specificity in genome-wide assays. A unique*-m* substring has exactly one perfect match on one of the strands of the genome while all other approximate matches must have more than *m* mismatches. We developed a pattern growth approach to systematically mine such unique*-m* substrings from a given genome. The search for unique*-m* substrings from genomes is performed as one data mining task so that the similarities among queries are utilized to achieve tremendous speedup. The runtime of our algorithm is linear to the sizes of input genomes and the length of unique*-m* substrings. In addition, the unique*-m* mining algorithm has been parallelized to facilitate genome-wide computation on a cluster or a single machine of multiple CPUs with shared memory.

## Figures and Tables

**Table 1:** Seven “species” and their “genome” sizes.

“species”	Genome size in bases	order of magnitude
Phi-X 174	5,386	10^3^
Human mitochondrion	16,569	10^4^
Mycoplasma genitalium	580,077	10^5^
Thermoplasma acidophilum	1,564,906	10^6^
Saccharomyces cerevisiae	12,156,320	10^7^
Caenorhabditis elegans	100,258,171	10^8^
Human	3,080,436,077	10^9^
